# A longitudinal MRI study on lymph nodes histiocytosis of a xenograft cancer model

**DOI:** 10.1371/journal.pone.0181043

**Published:** 2017-07-13

**Authors:** María Jiménez-González, Sandra Plaza-García, Janire Arizeta, Silvia Bianchessi, César Trigueros, Torsten Reese

**Affiliations:** 1 Magnetic Resonance Imaging Group, CIC biomaGUNE, San Sebastián, Guipúzcoa, Spain; 2 Metabolism Division, Johns Hopkins University, Baltimore, Maryland, United States of America; 3 Fundación Inbiomed, Hematopoietic and Mesenchymal Stem Cell lab, Guipúzcoa, Spain; 4 Mouse & Animal Pathology lab (MAPLab) Filarete foundation, Milano, Italy; University of South Alabama Mitchell Cancer Institute, UNITED STATES

## Abstract

**Background:**

Efforts are continuously made to detect and investigate the pivotal processes and interplay between the response of sentinel lymph node and malignant cells from a primary tumor. Conversely, some frequently used tumor animal models, such as human cancer xenografts, rarely feature metastasis. Therefore, lymph node alterations are seldom assessed. We consider that studying lymph node response could contribute to the understanding of host reaction to cancer. In the present study, we explored the presence of regional lymph node alterations in parallel with tumor growth using a pancreatic tumor xenograft model which does not develop metastasis.

**Methods and findings:**

We established an animal cancer model by the subcutaneous inoculation of PANC-1 (a metastatic human pancreatic cancer cell line) in the left upper flank of athymic nude mice. Tumor animals, along with controls (n = 7 / group) were subjected to Magnetic Resonance Imaging (MRI) in order to follow tumor growth and brachial and axillary lymph nodes alterations over several weeks. Further histological analyses were performed at the end of the study.

The individual average of the different lymph nodes sizes was 15–40% larger in the tumor animals compared to control animals at week 8 to week 20. The tumor size and lymph node size were not correlated. Histological analysis of the lymph nodes showed paracortical histiocytosis. No metastasis to lymph nodes could be detected by histology. In tumor bearing animals, histiocytosis was associated with isolated apoptotic bodies and migration of human tumoral cells was confirmed by specific immunostaining of human origin markers.

**Conclusions:**

The lack of metastasis as well as the pathological manifestation of the lymph node alteration in this pre-clinical model established here parallels findings in patients with sinus histiocytosis that is correlated with improved survival.

## Introduction

Several studies have tried to understand the mechanism of lymph node metastasis arising from solid tumors like breast [1, 2], pancreatic [3], cervical [4] and gastric cancer [5]. The lymph node structure harbours cell populations of the innate and adaptative immune system which, in principle, will have a capacity of tackling the metastatic spread [6, 7, 8]. In fact, lymph node activation with histiocytosis described in patients with several cancers, such as breast [9], head and neck [10] and bladder [11] was a good prognostic indicator for survival. On the contrary, lymphatic metastasis is an early event within the tumor progression that is considered a key factor in poor prognosis, especially after resection of the tumoral mass [12]. Thus, a common procedure for melanoma and breast cancer is to perform a sentinel lymph node biopsy in search for the presence of malignant cells which, in turn, may indicate the spread of cancer in other organs [13, 14, 15]. Hence, efforts were made to investigate the pivotal processes and interplay between the response of sentinel lymph node and malignant cells from the primary tumor [16].

Cancer research is supported by the use of animal models which aim to provide biological features that resemble the human disease. Xenografts of many established human cancer cell lines and from primary cancer tissue have been developed in immunodeficient mice, as these animals do not reject the engraftment. This methodology helps to study the progression of the tumor as well as serving as an *in vivo* model for therapeutics testing. However, several authors have described the differences between wild-type and immunodeficient mice strains of lymph nodes by histology [17, 18] and Magnetic Resonance Imaging (MRI) [19]. Among all immunodeficient mice, the nude mouse strains present a single-gene mutation at *Foxn1* (winged-helix/forkead transcription factor) gene that produces lack of functional thymus in homozygotes. The absence of thymus derived T-cells prevents xenograft rejection and thus the nude mouse is frequently used for generation of human cancer models [20]. Nevertheless, the natural killer cells population (NK cells), which has a key role in the innate immune response, is highly active [21]. Moreover, nude animals present a normal macrophage activity [22].

In the present study, we aimed to exploit the, commonly observed, sinus histiocytosis in the lymph nodes of nude mice in order to investigate the innate immune system response to primary tumor. Thus, a subcutaneous tumor xenograft model was established by using PANC-1, a metastatic human pancreatic cancer cell line [23, 24] in nude mice. The principal goal of the study was to examine the interplay between the primary tumor and the response of the innate lymph node immunity. The first aim of the study was to assess longitudinally by MRI the size of selected lymph nodes in tumor-bearing mice in comparison to control animals. Secondly, this study explored if any correlation existed between tumor and lymph nodes size over time, as described for melanoma and breast cancer [25, 26]. Finally, we characterized by histology the role of innate lymph node immunity function.

## Materials and methods

### Cell culture

The human pancreatic tumor cell line PANC-1 was obtained from the European Collection of Cell Cultures (ECACC, Sigma Aldrich). Authentication of PANC-1 cell line was performed by ECACC. Their testing regime includes Short Tandem Repeat (STR) profiling and standardized PCR-based authentication methods. Moreover, cells were not passaged for more than 6 months. PANC-1 cells were maintained as monolayer cultures in RPMI-1640 (Lonza, #12–702) supplemented with 10% FCS (Sigma-Aldrich, #F7524), 100 units/ml of streptomycin, 100 μg/ml of penicillin (Invitrogen, #15140–122) and 2 mM Glutamine (Lonza, #17-605E). Cultures were grown at 37°C in a humidified atmosphere of 5% CO_2_. Regular testing of the presence of *mycoplasma* was performed with the use of a commercial kit (Lonza, #LT-518).

### Xenograft tumor model

This study was carried out in strict accordance with the recommendations in the Guide for the Care and Use of Laboratory Animals of the European Union directive 2010/63/UE. Animal experiments were performed in CIC biomaGUNE, institution which hold a full accreditation from the Association for the Assessment and Accreditation of Laboratory Animal Care International (AAALAC). The study was authorized by the Basque Government (Guipuzkoako Foru Aldundia), Code: PRO-AE-SS-035. Mice were held in groups in individually ventilated cages and manipulated in safety cabinets with HEPA filters. Anesthesia treatment consisted of isofluorane administration 1.5–2.5% in 100% O_2_. Animal sacrifice was performed by cervical dislocation.

Preparation of cells for injection consisted of collecting them in a pellet by centrifugation and resuspension in Dulbecco’s Phosphate Buffered Saline (Lonza, 17-213F) at a concentration of 20x10^6^ cells/ml. The cell suspension was mixed with Matrigel (Becton Dickinson, #354.234) at 4°C at a 1:1 ratio and finally 200 μl of the mixture was used for each injection. Seven seven-week old male CD-1 athymic-nude *Foxn1* nu/nu mice (Charles River) were injected subcutaneously with 2 million PANC-1 cells in the upper left flank, approximately 1–2 cm below the armpit. For the monitorization of the animals, we weighted them and we measured the tumor volume several times a week with a calliper. We calculated the volume using the formula (L x W^2^)/2 where L is the length (longest diameter of the tumor) and W is the width (longest perpendicular diameter with respect to L).

Seven naïve 7-week old male CD-1 athymic-nude Foxn1 nu/nu mice animals served as controls.

### MRI acquisition protocol

Measurements were made on an 11.7 Tesla horizontal bore Bruker Biospec 117/16 scanner (Bruker). The MRI experiments were performed at weeks 8, 14, 16 and 20 after PANC-1 cells inoculation with the use of a 40 mm transmit/receive mouse body volumetric coil. Animals were anesthetized prior to imaging using 3.5% isoflurane and maintained at 1.5–2.5% in 100% O_2_. Mice were placed in a MRI compatible cradle and kept warm throughout the image acquisition using a heated water blanket. Rectal temperature and respiration rate were monitored with an MRI compatible animal monitoring system (SA Instruments Inc.). The MRI session started with a fast scout scan using an Intragate FLASH sequence (Bruker, Ettlingen, Germany). The pilot scan consisted of five 1 mm thick slices in axial, sagittal and coronal orientations and was run for 1 min 21 s. Afterwards, MR imaging was performed using a gradient echo sequence with the following parameters: Respiration synchronized (TR = 436.5 ms), TE = 3 ms, Flip Angle = 60 degree, Field of View = 30 mm x 30 mm, Matrix = 256 x 256, Slice Thickness = 0.6 mm, n° of continuous Slices = 20 and NA = 2. The spatial resolution of the T1-, weighted scan was 117 x 117 x 600 μm^3^ with a total acquisition time of 6–9 min (depending on the respiratory rate)

As the regions of interest in this study were the tumor and the axillary and brachial lymph nodes, we acquired two packages, one for the tumor and the other for the lymph nodes. Total time acquisition for each session took less than 1 hour.

### Imaging analytics

The volumes of the tumors and lymph nodes were obtained manually by one reader. Paravision 5.1 was used to draw the regions of interest in each of the slices that covered the individual lymph nodes or tumor. The sum of the individual areas and subsequent multiplication by the slice thickness gave rise to the total volume.

### Immunohistochemistry

After the last imaging session we sacrificed the animals. Subcutaneous tumors, the right and left brachial lymph nodes and the right and left axillary lymph nodes were extracted. The specimens were fixed with 10% neutral buffered formalin (Sigma-Aldrich, #HT501128) and subsequently embedded in paraffin. Selected 5 μm thick slices were prepared and then stained by hematoxylin and eosin (H&E) and immunostaining assays:

A determination for human origin cells was performed by the use of a human-specific monoclonal antibody (Millipore #MAB1273, Clone: 113–1) directed against a 65-kDa nonglycosylated protein component of human mitochondria (hMit) exclusively found in human cells. Before incubation, slides were pretreated in preheated (95–100°C) citrate buffer (10 mM, pH 6.0) and heating in a microwave during 15 min for antigen retrieval. Then, tissue sections were incubated overnight with the antibody against hMit. Subsequently, sections were incubated with biotinylated secondary antibody and further revealed with the chromogen 3,3’-diaminobenzidine (DAB). Finally, slides were counterstained with H&E.

For the identification of histiocytes/macrophages and the presence of cells of human origin, serial sections from each sample were immunostained with a rabbit polyclonal antibody against Iba1 (Wako) or with a rabbit monoclonal antibody against MHC-I (Abcam), respectively. Sections were incubated with a biotinylated secondary antibody (goat anti- rabbit, VC-BA-1000-MM15, Vector Laboratories). Sections were then labeled by the avidin-biotin-peroxidase complex (ABC) procedure with a commercial immunoperoxidase kit (VC-PK-6100-KI01, Vector Laboratories). The immunoreaction was visualized with DAB (VC-SK-4100-KI01, Vector Laboratories) substrate and sections were counterstained with Mayer’s hematoxylin (C0302, Diapath).

### Statistical analysis of tumor and lymph nodes

Analysis of the differences between means of control and tumor bearing mice groups were performed using a Student t-test (two tailed, unpaired). The analysis within the same group in longitudinal studies was performed using ANOVA test. Signification levels for correlation coefficients were assessed for one-tailed test in normally distributed samples. P values below 0.05 were considered significant.

## Results

### Subcutaneous tumor analysis

**Fig 1:**
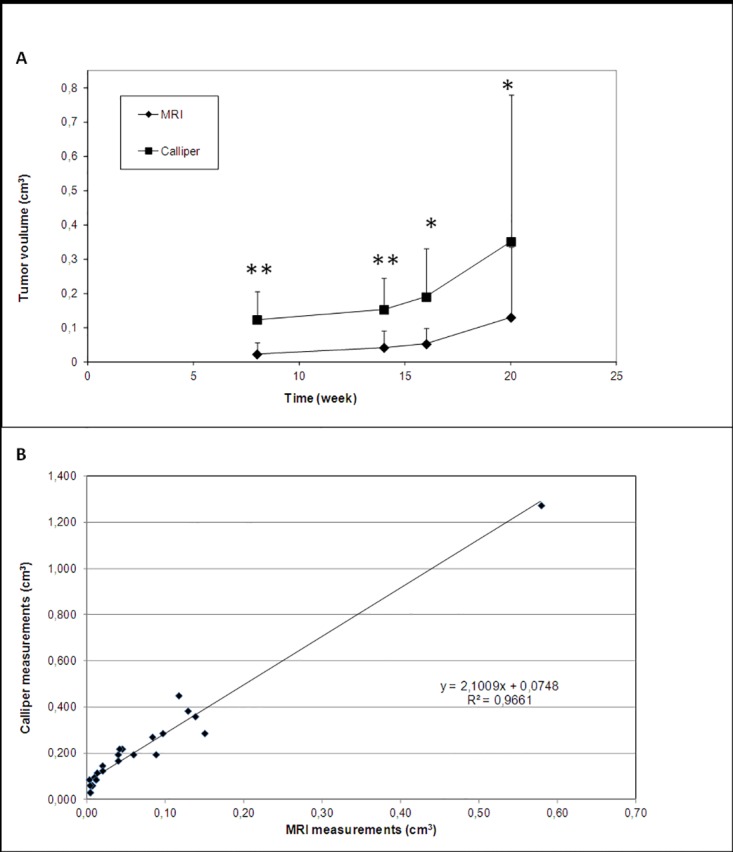
Tumor size over time. (A) Tumor growth profile measured by Caliper and MRI. *Points*, mean; *bars*, SD *p < 0.05, **p < 0.01. (B). Correlation between the two measurements, each point represents caliper measurement of tumor size (in cm^3^) plotted against MRI measurement of the same tumor specimen (n = 7), assessed 4 times over 20 weeks.

The average growth rate of the tumors accelerated over time and showed an exponential-like curve, as measured by caliper or by MRI (Fig 1A). We could observe a significant correlation (r = 0.966, p < 0.005) between the two measurements (Fig 1B) with caliper values being significantly higher. This can be easily understood as the caliper measurement also included non-tumor tissue (e.g. skinlayers, fat.).

### Tumor morphology by MRI and histology

**Fig 2:**
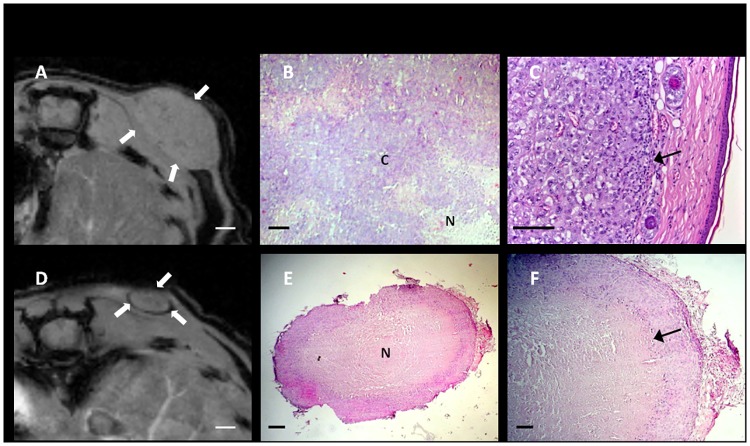
Representative MR images and H&E stained tumor samples. A growing tumor (A-C) and a non-growing tumor specimen (D-F). White arrows are pointing to the tumor mass, some areas show inflammatory infiltration (black arrows) N = Necrotic tissue, C = Carcinoma. *Bar* in (A) and (D), 1 mm. *Bar* in (B), (C) and (F), 100 μm. *Bar* in (E), 250 μm.

Magnetic Resonance images showed tumors with a scarce internal heterogeneity (Fig 2A). Histological H&E staining revealed areas of solid undifferentiated epithelial carcinoma tissue (Fig 2B). Dispersed necrotic areas as well as some tumoral mass infiltrating musculature and the presence of inflammatory cells in the periphery could be detected (Fig 2C). Interestingly, several non-growing ‘ring-like’ tumors were seen in MR images (Fig 2D). Histology showed that they corresponded to nodular structures in which there was a thin layer of tumoral tissue at the periphery and a wide central core of necrotic tissue (Fig 2E and 2F).

### Lymph nodes enlargement in tumor bearing animals

The lymph nodes volumes were derived from MR images. The nodes neither grew significantly in tumor-bearing animals, from 8 to 20 weeks after PANC-1 inoculation, nor in the age matched controls (one-way ANOVA single factor analysis). No correlation between lymph node size and animal weight was found (p > 0.05 for both tumor bearing and control animals).

**Fig 3:**
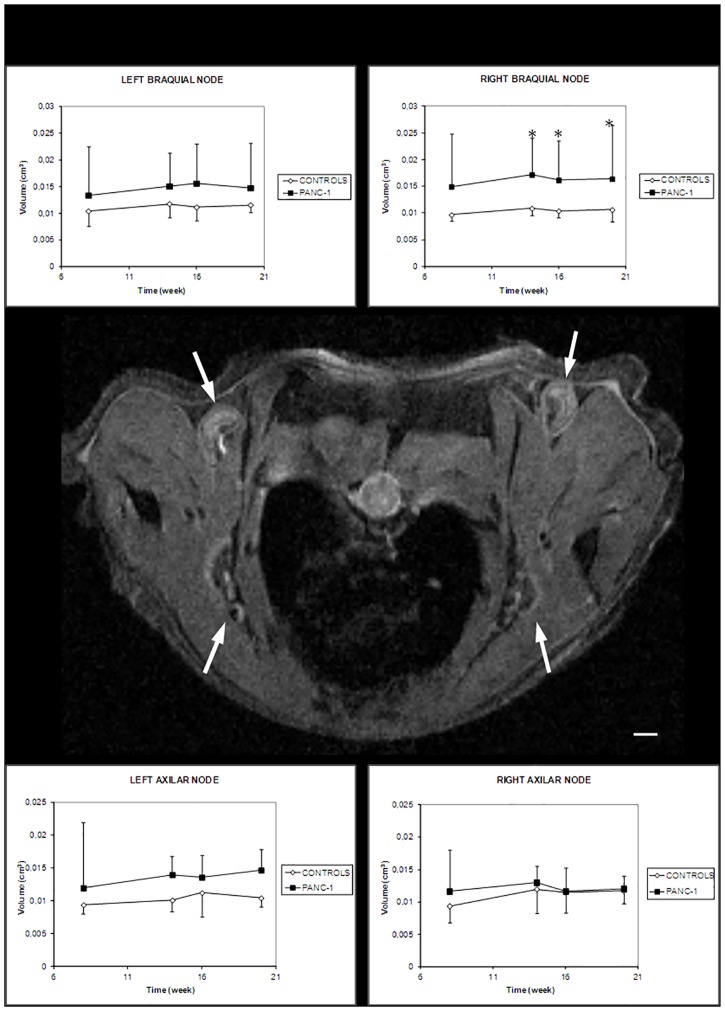
Lymph nodes volumes measured longitudinally by MRI (right and left brachial and right and left axillary) in PANC-1 tumor and control mice. In the middle, representative T_1_- weighted image of the analyzed lymph nodes in a control mouse. (n = 7/group). *Points*, mean; *bars*, SD. *, p < 0.05 (unpaired, one-tail Student’s test). *Bar*, 1 mm.

The average volumes of the different lymph nodes in the tumor bearing mice were 15–40% larger compared to the control ones, except for right axillary node at weeks 16 and 20 (Fig 3), the lymph node furthest away from the tumor location. The trend in lymph node volumes between the two groups did not reach significant levels except for right brachial nodes, where the difference was largest and thus reached significant values at weeks 14, 16 and 20 post injection (p = 0.042, p = 0.015 and p = 0.047 respectively).

**Fig 4:**
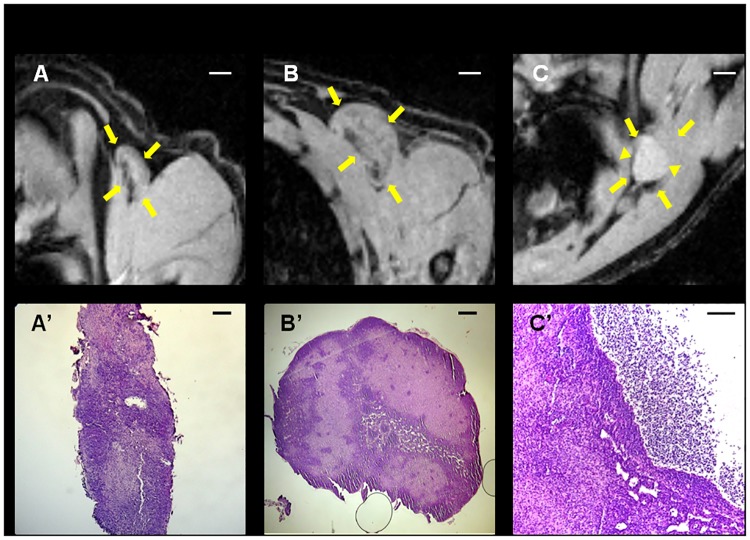
MR images of different lymph node ‘types’ (see arrows) and the correspondent H&E histology. (A, A’) Brachial node, normal structure and size. (B, B’) Brachial node, normal structure and large size. (C, C’) Wall of cyst (left) surrounding a large cavity that contains a serocellular fluid (right) in a swollen axillary node (triangles in C). *Bar* in (A), (B) and (C), 1 mm. *Bar* in (A’) and (B’), 250 μm. *Bar* in (C’), 100 μm.

There was no significant correlation between tumor size and the analysed lymph nodes sizes at any measured time-point with the exception of right brachial node and tumor size at week 20 (Table 1). Some of the animals presented a bright rounded dot located in the medullar part of the lymph node which was observable by MRI; in several cases the lymph node was enlarged and seemed ‘swollen’ with the normal structure completely lost (Fig 4C), a feature that has been already described by other authors [19].

**Table 1:** Values for coefficient of correlation between tumor and selected lymph nodes overtime (n = 7/group).

Coefficient of Correlation (R)	W8	W14	W16	W20
PANC-1 Tumor vs Right Brachial Node	0.117	0.104	0.408	0.815*
PANC-1 Tumor vs Left Brachial Node	0.240	0.216	0.041	0.579
PANC-1 Tumor vs Right Axillary Node	0.007	0.416	0.101	0.054
PANC-1 Tumor vs Left Brachial Node	0.084	0.099	0.315	0.046

*; p < 0.05

### Presence of human apoptotic bodies and migrating cancer cells in the lymph nodes

**Fig 5:**
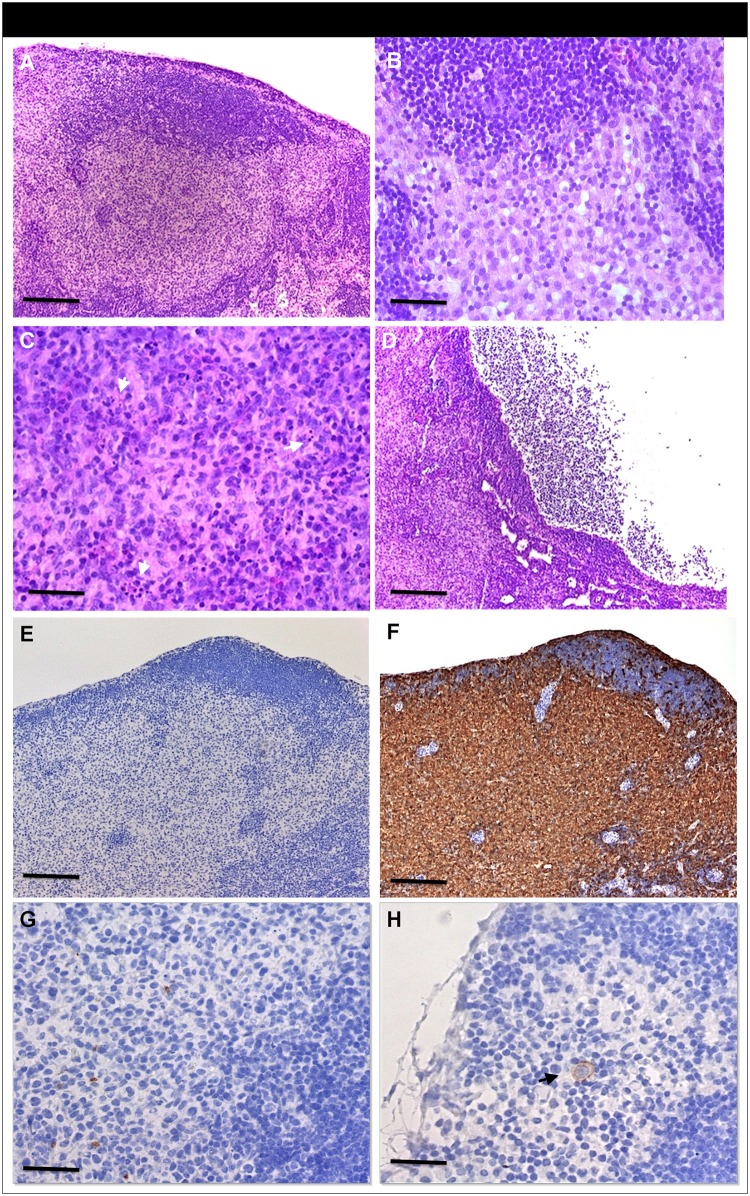
Representative histological and immunohistochemical findings of lymph nodes. H&E staining which presents paracortical histiocytosis (A, B). Presence of apoptotic bodies (C, white arrows) and cyst (D). Histiocytes/macrophages (E) control and (F) Iba1staining showing a strong positivity in the paracortical area. Human cells (MHC-I) staining (G, H) showing scattered positive fragmented cells (G) and a single human intact cell located in the subcapsular sinus (H, black arrow), in tumor bearing animals only. *Bar* in (A), (D), (E), (F); 200 μm. *Bar* in (B), (C), (G), (H); 50 μm.

In order to elucidate the nature of the alterations found in the lymph nodes of control and tumor-bearing mice, morphological features were described in hematoxilin-eosin stained samples by a pathologist (Fig 5). Lymph nodes were characterized by partial loss of normal structure (Fig 5A). The cortex was atrophic and the paracortical area lacked T- lymphocytic cells (which can be expected in nude mice). Paracortical areas were filled by histiocytic cells with large eosinophilic cytoplasm. A large number of histiocytic cells were also detected in the medullary area. The ‘swollen’ nodes presented medullary cysts which, in some cases, filled the whole medullary compartment. Only in tumor bearing animals we appreciated the presence of apoptotic bodies characterized by the condensed, irregular nuclei shown in H&E (Fig 5C). The presence of metastasis could be excluded in all cases.

**Fig 6:**
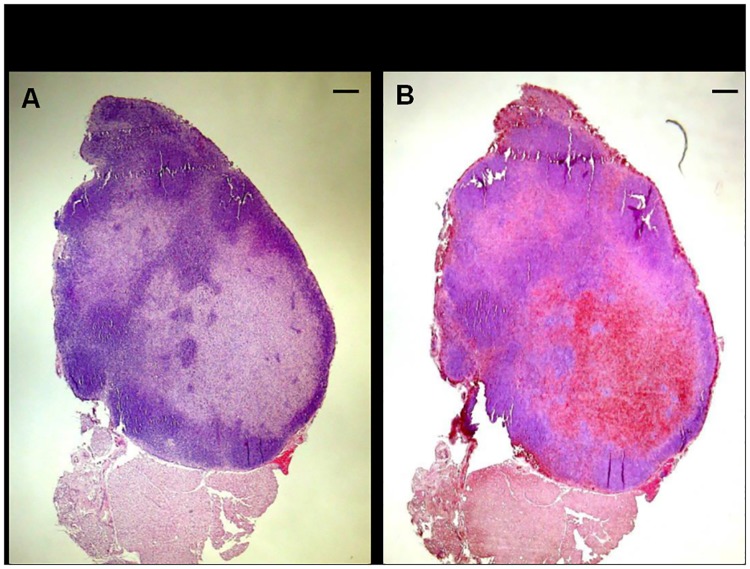
Representative images showing the positive staining for exclusive human mitochondria epitope (hMit). Low density areas of histiocytosis of an axial lymph node (B) and its corresponding H&E control (A). *Bar* in (A) and (B), 250 μm.

As a screening test for finding potential cells of human origin, we performed a specific staining for an exclusive epitope of the human mitochondria (MAB1273). The results reflected the positive staining of a defined area corresponding to paracortex/medulla which, as well, presented histiocytosis (Fig 6).

Additionally, we performed specific immunostaining for the identification of the nature of cells: Histiocytes/macrophages (Iba1) and human origin cells (MHC-1) comparing lymph nodes of control and tumor-bearing animals. Paracortex and medullary area of the lymph nodes strongly stained for histiocytes/macrophages in both groups (Fig 5F). These cell types occupied the paracortex area which non-existing T-lymphocytes leave vacant. Iba1 staining is negative in the follicles, where B-cells reside.

On the other hand, MHC-1 positive staining was appreciable in the lymph nodes of PANC-1 tumor-bearing animals only. These immunostainings were seen as scattered dots associated with apoptotic bodies within the areas of histiocytosis (Fig 5G). Another remarkable finding was the detection of a single positive intact tumoral cell in the subcapsular sinus, next to the afferent lymph vessel (Fig 5H). In summary, it implies that in this model the primary tumor cells preferentially migrate via the lymphatic system and not via the blood.

## Discussion

Our T1-weighted protocol was optimised to image the tumor size only. Initial experiments were also performed with a T2-weighted sequence and the relaxation times were determined, however, and T1- and T2 values are homogeneous throughout the tumor [27]. The MR images of the tumors were homogeneous in appearance, hardly capable of distinguishing between necrotic core and tumor mass. Additional characterization of the tumor itself could be performed using diffusion weighted MRI [28, 29], dynamic contrast enhanced MRI [30] or the perfusion status could be determined using arterial spin labelling methodologies [31]. As expected, the tumor volume measured by MR was significantly lower than measured by caliper (which is also measuring the skin). Three lymph node ‘types’ could be generally identified by MRI. First, the lymph node appeared normal and was able to be visualized with an in-plane resolution of < 117 μm at 11.7 Tesla. Second, the lymph node was larger but displayed roughly a morphology similar to a normal lymph node (cortical sinus, medullar sinus and follicles). Third, the lymph node appeared swollen and bright on MR images which showed a medullary cyst within the lymph node. The cysts occupied almost the complete area from medullar sinus to paracortex, displacing follicles and cortex. Similar features have been reported in other strains [17, 19] and athymic rats [18]. The SCID mouse, which lacks complete adaptative immunity (B and T-Cells), had on average only 25% of the wild type lymph node size [19]. Paradoxically, the nude mouse, which lacks mature functional T-Cells, displayed much larger lymph nodes than the wild type. It seems that the nude strains compensate for the impaired immune system by lymph nodes hyperplasia. Interestingly, subcutaneous xenograft cancer models are commonly utilizing this athymic immunodefficient mouse. Nevertheless, despite implantation of aggressive human cancer cell line, frequently derived from patient that suffered lymphatic metastasis, an invasion to the lymph node is seldomly observed [32, 33].

The capacity of active lymph nodes to tackle the metastatic spread of a primary tumor is not fully understood. Thus, we implemented a PANC-1 subcutaneous tumor model in athymic mice in order to elucidate the role of lymph node activation. We selected PANC-1 as it is a human tumoral cell line derived from a patient who suffered from lymph node metastasis [23]. As predicted, no lymphatic metastasis into the surrounding lymph nodes was found in our subcutaneous xenografts. However, lymph nodes of PANC-1 inoculated animals were larger than control ones. It is described that a lack of lymphocytes in athymic nude mice creates space for the proliferation of histiocytes [19]. In both groups, control and tumor bearing animals, histiocytosis occurred; however, the even bigger lymph node sizes in tumor animals could indicate an increased activation of these histiocytes in the presence of the tumor. Whereby the lymph nodes grew between 14 and 26 weeks of age, the size ranked similar over the complete study duration. Thus, large lymph nodes at week 14 were also large at week 26 whereby the growth curve of the tumours followed an exponential pattern. Therefore, the manifestation of a general immune response leads to a substantial increase in regional lymph nodes that is not correlated to the tumour volume. These findings may imply a variability of innervations of afferent lymphatic vessel towards the regional lymph nodes. Further experiments covering earlier stages of the tumor progression are needed for elucidating the initial steps that leads to the presence of larger lymph nodes in tumor animals.

A direct innate immune reaction to tumoral cells may cause histiocytes to replicate in large numbers, which in turn is causing an overall size increase of the regional lymph node. A persistent delivery of proliferating isolated cells from the primary tumor side to the regional lymph node was also observed by fluorescent dissecting microscopy in a human gastric carcinoma subcutaneous model, whereby the proliferation was terminated by surgical removal of the primary tumor [34]. In our study, it is apparent that, despite migrating human cancer cells reaching the lymph node sinus, potentially in high frequency, no metastasis is formed. The mechanisms to tackle malignant cell dissemination and trigger subsequent apoptotic cell death within the lymph nodes are still unknown. Earlier studies point towards adhesion molecules on the surface of the cancer cells and the endothelium of the sinus being responsible for the cancer cell arrest and/or entrapment [35, 36, 37]. Additionally, NK cells might play a role in attacking single proliferating cells [33, 34]. Another possible mechanism refers to entrapment of the cancer cells by reticular fibers [38]. Our histological analysis showed the presence of apoptotic bodies only in the tumor bearing mice. Specifically, we were able to detect MHC-1 staining of fragmented structures which could be referred as apoptotic bodies from human cancer cell origin. A similar positive staining was found for human mitochondria epitope (hMit), in a much higher amount than the MHC-1 signal. As both staining were localized in histiocytosis regions, we may hypothesize that, in the process of human cell apoptosis and posterior engulfment of fragments by phagocytic cells, membrane MHC-1 molecule is rapidly degraded whereas mitochondria structure is still present in phagocytosis [39]. These findings support that the isolation of the proliferating cells by histiocytes in general might be responsible for the cascade that ultimately lead to apoptotic cell death within a regional infiltrated lymph node. Specifically, macrophage entrapment was previously perceived to be responsible for induced cell death within the lymph node sinus [40]. The encapsulation of tumor proliferating cells by histiocytes appears to result in a shielding from the lymph node environment and as such builds an isolated cell cluster. As the cancer cells and histiocytes in this substructure are in close contact with each other, it is likely that ‘certain signals’ are exchanged. Isolating the cancer cell macroscopically from the lymph node environment might deprive cancer cells of essential survival factors like vascular endothelial growth factor-C/D [41, 42] and plateled derived growth factors [43]. Alternatively, the histiocytes simply prevent proliferating cell clustering and settling which might be mandatory for replication of the tumoral cells. The side of immune reaction is located within the marginal sinus of the afferent lymph node, regularly associated with ED3+ macrophages that are capable of incorporating foreign bodies [44]. As intact or apoptotic cancer cells and bodies are sparsely distributed within the lymph node in our model, no metastases were observed. This is similar to the initial phase (several hours) but is in stark contrast to a metastatic model in immunocompetent Donyro rats, where AH130 rat ascites hepatoma cells overcame the initial anti-metastatic defense [16]. In their model, four days after injection, the proliferating cell populated the complete marginal lymph node, deprived the sinus of histiocytes and metastasized into the medullar sinus. Our findings potentially suggests that the immune system might be capable of coping only with a certain number of cells or foreign bodies, with a large number of histiocytes required to isolate the cancer cells to trigger apoptosis. Importantly, the lymph node functions as a barrier [45] and prevents, via this unexplained mechanism, metastasis without having to rely on adaptive immune response or antibody presentation. The defence can be overrun if the histiocytes are outnumbered within the marginal sinus and following the establishment of a metastasis; the system appears to be losing the capability of self cleansing. Nevertheless, our experimental results are not sufficient to explain the specific role of histiocytosis or whether its presence is a direct signal of anti-cancer defense. Importantly, other mechanism and factors are to be studied: grade of histiocyte proliferation, nature of cells (NK, macrophage, mastocytes), etc.

Features of the primary tumor cells can establish different patterns for dissemination, invasion and immunological evasion. The aberrant expression of proteins, such as mucins, could be playing an important role in the immunosuppression that facilitates cancer spreading [46]. As an example, MUC16 expression has been detected in various pancreatic cell lines derived from metastasis (Capan1, Colo 357, T3M4), meanwhile its expression was negative in Panc-1 cell line, derived from primary tumor [47]. However, as shown here, the PANC-1 model can serve as a chronic pre-clinical model that possesses migrating Panc-1 tumor cells to sentinel lymph node, without causing metastasis. Hence, future *in vivo* studies with other characterized pancreatic cell lines could shed light onto the importance of proteins on cancer-cell migration and immune system interaction.

Taken together, the current manuscript and many other studies' conclusions strengthen the importance of lymph nodes staging for an accurate prognosis evaluation, i.e. definition of the ratio between metastatic vs non-metastatic lymph nodes [48].

In summary, the preclinical model described here, a subcutaneous PANC-1 tumor in a nude mouse strain, has no metastatic mass but with tumoral apoptotic cancer cells from the primary tumour side to regional lymph nodes. Ultimately, as the model resembles a form of sinus histiocytosis it may serve as a chronic model to:

- Study the possible role of innate immune system cancer cell recognition [49] as well as immune-tolerance [50].
- Investigate in-depth cancer cell—histiocyte messaging with the goal to derive new biomarker or putative targets for cancer management and/or treatment.
- Clarify important aspects of the underlying mechanisms and signalling that are responsible to stop proliferating cancer cells from building active colonies in form of lymph node metastasis.
- Study nutritional and environmental aspects as well as beneficial and detrimental effects of novel treatments that may modulate histiocytosis, primary tumor cell migration and lymph node capacities.
- Analyse the influence of therapy, radiotherapy and sentinel lymph node resection on metastatic spread.
- Scrutinse if the relation between the lack of metastasis and histiocytosis is incidental or causal.

## Conclusion

The pre-clinical model described here, possesses migrating Panc-1 tumor cells to sentinel lymph nodes, without causing metastasis. The model resembles a form of sinus histiocytosis and is suitable to study important aspects of the underlying mechanisms and signalling that are responsible to stop proliferating cancer cells from building active colonies in form of lymph node metastasis. Hence, future *in vivo* studies could be used to identify novel imaging- or bio-markers describing the interplay of cancer-cell migration, sentinel lymph node pathophysiology and anti-metastatic effects. Lymph node activation, directed by innate immune system, could be related and/or partially imply that primary tumor cells preferentially migrate via the lymphatic system and not via the blood. It may explain why patients with resected tumors but sinus histiocytosis have a better prognosis than patients without these ‘reactive’ lymph nodes.
